# Antiviral Activity of Reagents in Mouth Rinses against SARS-CoV-2

**DOI:** 10.1177/0022034520967933

**Published:** 2020-10-22

**Authors:** F. Carrouel, L.S. Gonçalves, M.P. Conte, G. Campus, J. Fisher, L. Fraticelli, E. Gadea-Deschamps, L. Ottolenghi, D. Bourgeois

**Affiliations:** 1 University Claude Bernard Lyon 1, Laboratory “Systemic Health Care,” University of Lyon, Lyon, France; 2 Faculty of Dentistry, Estacio de Sá University, Rio de Janeiro, Brazil; 3 Department of Public Health and Infectious Diseases, Sapienza University of Rome, Rome, Italy; 4 Department of Restorative, Preventive and Pediatric Dentistry, Faculty of Dental Medicine, University of Bern, Bern, Switzerland; 5 THEnet, Training for Health Equity Network, New York, NY, USA; 6 RESCUe-RESUVal Network, Lucien Hussel Hospital, Vienne, France; 7 Emilie Roux Hospital Center, Le Puy-en-Velay, France; 8 Department of Oral and Maxillo-Facial Sciences, Sapienza University of Rome, Rome, Italy

**Keywords:** COVID-19, mouthwashes, saliva, oral, viral load, clinical trial

## Abstract

The oral cavity, an essential part of the upper aerodigestive tract, is believed to play an important role in the pathogenicity and transmission of SARS-CoV-2. The identification of targeted antiviral mouth rinses to reduce salivary viral load would contribute to reducing the COVID-19 pandemic. While awaiting the results of significant clinical studies, which to date do not exist, the commercial availability of mouth rinses leads us to search among them for reagents that would have specific antiviral properties with respect to SARS-CoV-2. The challenges facing this target were examined for 7 reagents found in commercially available mouth rinses and listed on the ClinicalTrials.gov website: povidone-iodine, chlorhexidine, hydrogen peroxide, cyclodextrin, Citrox, cetylpyridinium chloride, and essential oils. Because SARS-CoV-2 is an enveloped virus, many reagents target the outer lipid membrane. Moreover, some of them can act on the capsid by denaturing proteins. Until now, there has been no scientific evidence to recommend mouth rinses with an anti–SARS-CoV-2 effect to control the viral load in the oral cavity. This critical review indicates that current knowledge of these reagents would likely improve trends in salivary viral load status. This finding is a strong sign to encourage clinical research for which quality protocols are already available in the literature.

## Introduction

Considering mouth rinses as agents that can reduce the viral load of severe acute respiratory syndrome coronavirus–2 (SARS-CoV-2) in the fight against the COVID-19 pandemic is an extremely attractive concept (Carrouel et al. 2020; O’Donnell et al. 2020). Logically, this concept should lead to in vivo studies and clinical recommendations. If so, it would be strategic to contribute to the development of a text based on the idea of therapeutic oral biofilm flushing for COVID-19 that would introduce new ways of thinking and new ways of working around oral care for the dental profession and the general public. Beyond COVID-19, it would also be an entry point with the medical and health care community to continue to emphasize the importance of the oral sphere in the transmission of viruses and in the fight to reduce the transmission of infectious diseases. History reinforces that outbreaks such as H1N1, SARS, and Middle East respiratory syndrome (MERS) are not isolated, once-in-a-lifetime events. Rather, we need to prepare for rapidly emerging epidemics of respiratory viral origin and need a new generation of products, technologies, and techniques that are able to respond in an agile and multidisciplinary manner.

The use of an antiviral mouth rinse during oral care has been recommended by some national dental authorities to protect dental personnel and patients, but there are currently no recommendations from the Ministries of Health or the World Health Organization (WHO) for the use of mouth rinses in patients with COVID-19 or with respect to preventive measures at a population level (Alharbi et al. 2020; Ather et al. 2020). Available guidance is not based on evidence of the clinical efficacy of preprocedural mouth rinses to reduce SARS-CoV-2 viral loads or to prevent transmission but rather on the clinical efficacy of mouth rinses on similar viruses, such as SARS-CoV. It is imperative that research address this gap in knowledge.

The concept put forward is that some commercially available mouth rinse formulations may play a role in reducing the transmission of SARS-CoV-2 and may be useful in the current pandemic (Carrouel et al. 2020; O’Donnell et al. 2020). For health and dental services, this information might be of value for dentists to reduce the exposure of their patients and the risk of contamination. This thinking should go beyond the spatially and temporally limited phase. If antiviral mouth rinses kill the virus coming in, it follows that they would kill the virus going out. This would allow a set of broader recommendations that could be extended to clusters, communities at risk, health professionals, and the general population to reduce and eventually prevent the risk of transmission.

This critical review describes the existing body of evidence supporting the potential therapeutic effects of mouth rinse ingredients in preventing the transmission of SARS-CoV-2. The results of this review are based on in vitro and in vivo studies. In silico research based on computer-based virtual screening of SARS-CoV-2 has also been identified.

## The Oral Cavity as an Actor in the Spread of SARS-CoV-2

Because the oral cavity is an important reservoir of SARS-CoV-2, the use of an antiviral mouth rinse could be important in the fight against the COVID-19 pandemic. Indeed, SARS-CoV-2 is found in nasopharyngeal secretions, and its viral load is consistently high in the saliva, mainly in the early stage of the disease (Yoon et al. 2020). It is detected in 91.7% of saliva samples from COVID-19 patients, and the number of infective copies/mL can reach up to 1.2 × 10^8^ (To et al. 2020).

In addition, saliva is an important source of transmission during the COVID-19 pandemic. When a person coughs, sneezes, breathes, or converses, he or she produces saliva droplets containing microorganisms (Baghizadeh Fini 2020a). The quantity and the size of saliva droplets differ between individuals; therefore, the risk of transmission also varies. One cough or 5 min of conversation produces approximately 3,000 saliva droplets. One sneeze produces approximately 40,000 saliva droplet nuclei that can be disseminated several meters in the air (Baghizadeh Fini 2020a). Saliva droplets (>60 μm) allow the transmission of SARS-CoV-2 when persons are in close contact (1 m and 3 m; National Academies of Sciences, Engineering, and Medicine 2020). Moreover, even if it is not yet clearly established, virus-laden aerosols (droplets <60 μm) can contribute to the spread of SARS-CoV-2 and allow contamination at a distance of up to 7 to 8 m (Jayaweera et al. 2020).

Droplets containing SARS-CoV-2 penetrate in a host through the mouth or eyes or can be inhaled directly into the lungs. Thus, the host is infected and can then develop clinical signs of COVID-19 disease (Baghizadeh Fini 2020a, 2020b; Xu et al. 2020).

SARS-CoV-2 is an enveloped, single-stranded RNA virus. To act as a pathogen, the spike protein of SARS-CoV-2, activated by proteases, binds to its receptor, angiotensin-converting enzyme 2 (ACE2; Shang et al. 2020). ACE2 and a proprotein convertase furin, both involved in viral penetration into cells, are highly expressed in the salivary glands (Zupin et al. 2020). At the eye level, ACE2 and TMPRSS2, a cell surface–associated protease that facilitates viral entry following binding of the viral spike protein to ACE2, are expressed on the human ocular surface (Zhou et al. 2020). After this attachment to the cell surface, the viruses enter endosomes, and in some cases, the viral and lysosomal membranes fuse (Shang et al. 2020).

## Mouth Rinses to Prevent the Dissemination of SARS-CoV-2

To decrease the risk of transmission of SARS-CoV-2 by COVID-19 patients, the viral load from the oral cavity must be decreased. One of the most efficient actions for this is the use of an antiviral mouth rinse (Carrouel et al. 2020; Herrera et al. 2020). Reviews of the literature concluded that mouth rinses containing cetylpyridinium chloride (CPC) or povidone-iodine (PVP-I) can decrease the severity of COVID-19 by reducing the SARS-CoV-2 oral viral load and can decrease the risk of transmission by reducing viral load in droplets generated in normal life or in aerosols produced during dental procedures (Herrera et al. 2020; Kumar et al. 2020). In addition, if we generate a nonexhaustive list of mouth rinses that are marketed as containing antiviral molecules (Table 1), we notice that other compounds could be of interest in the fight against SARS-CoV-2, such as hydrogen peroxide (H_2_O_2_), chlorhexidine (CHX), cyclodextrin (CD), Citrox, or essential oils (EOs) (Carrouel et al. 2020; Herrera et al. 2020).

**Table 1.** Product Information of the Main over-the-Counter Oral Rinses or Mouth Rinses according to Their Active Ingredients.

Active Ingredient	Package Name	Strength	Manufacturer Name
Chlorhexidine (187 studies^a^)	Paroex^®^	0.12%	Sunstar Americas, Inc., USA
	Perio-Aid^®^ Intensive Care	0.12%	Dentaid SL, Spain
	Kloroben^®^	0.12%	Kloroben, Turkey
	Corsodyl Care^®^	0.20%	SmithKline Beecham Consumer Healthcare, UK
	Periogard^®^	0.12%	Colgate Oral Pharmaceuticals, Inc., USA
	Curasept^®^	0.20%	Curasept S.p.A., Italy
	Peridex™ Oral Rinse	0.12%	3M™ Espe Dental Products, USA
	Eludrilpro^®^	0.10%	Pierre Fabre Oral Care, France
	Avohex^®^ Gluconate	0.20%	Middle East Pharmaceutical Industries Co. Ltd., Saudi Arabia
	Acclean Oral Rinse	0.12%	Xttrium Laboratories, Inc, USA; Henry Schein, Inc, USA
	Chlorhexidine Gluconate 1	0.12%	^b^
Cetylpyridinium chloride (15 studies^a^)	Crest^®^ Pro-Health^®^ Multi-Protection	0.07% to 0.1%	The Procter & Gamble Manufacturing Company, USA
	Perio·Aid^®^ Intensive Care	0.12%	Dentaid SL, Spain
	Colgate Total—Colgate Zero	0.025%, 0.075%	Colgate-Palmolive Company, USA
	Assured Fresh Mint Oral Health Rinse	0.07%	Greenbrier International, Inc., USA
Citrox (1 study^a^)	Curaprox Perio Plus Regenerate^®^	0.01%	Curaden AG, Switzerland
Cyclodextrin (1 study^a^)	Curaprox Perio Plus Regenerate^®^	0.1%	Curaden AG, Switzerland
Essential oils (25 studies^a^)	Listerine Professional Gum Therapy^®^		Johnson & Johnson, USA
	Listerine^®^ Zero™		Johnson & Johnson, USA
	Cool Mint Listerine^®^		Johnson & Johnson, USA
	Decapinol^®^		Sinclair Pharma Ltd., UK
Hydrogen peroxide (8 studies^a^)	Crest^®^ Oral-B Mouth Sore Mild Mint	1.5%	The Procter & Gamble Manufacturing Company, USA
	3D White™ Glamourous White Multi-care Whitening	>1%	The Procter & Gamble Manufacturing Company, USA
	Colgate^®^ Peroxyl^®^ Mouth Sore Rinse	1.5%	Colgate Oral Pharmaceuticals, Inc., USA
	Oral B^®^ Mouth Sore Special Care Rinse	1.5%	The Procter & Gamble Manufacturing Company, USA
	Sore Mouth Cleanser^®^	1.5%	Vi-jon, Inc., USA
	Medline^®^ Rinse	1.5%	Medline Industries, Inc., USA
	Perox-a-mint Solution	1.5%	Sage Products LLC, USA
	Peroxy Shield Mouth Sore	1.5%	Dental Technologies, Inc., USA
Povidone-iodine (1 study^a^)	Betadine^®^	1%	Pfizer Ltd., USA
	Halodine^®^ Oral Rinse	1.7%	Halodine LLC, USA
	Povidone Iodine Gargle^®^	0.5%	Humco Holding Group, Inc., USA
	Betadine Gargle	0.5%	Avrio Health L.P., USA

This table is a synthesis of data obtained from electronic research organized in the databases ClinicalTrials.gov, Drug Information Portal (National Institutes of Health), National Drug Code List, and PubMed. The following MeSH and non-MeSH search terms were used to encompass every type of over-the-counter (OTC) mouthrinse or mouthwash: (“mouth rinse” [MeSH terms] OR “mouthwashes” [All fields]) AND (“Chlorhexidine” [MeSH terms] OR (“Cetylpyridinium chloride” [All fields] OR “essential oils” [All fields] OR “hydrogen peroxide” [All fields] OR “Povidone-iodine” [All fields] OR (“Citrox” [All fields] OR “Cyclodextrin” [All fields]). When the product name was missing, contact by e-mail or ResearchGate (www.researchgate.net) was made with the principal investigator. This list of OTC drugs is not exhaustive.

a Number of clinical studies identified in ClinicalTrials.gov.

b Twenty-four OTC drugs use the same package name. The list of manufacturers’ names is available at https://ndclist.com.

**Figure 1.**
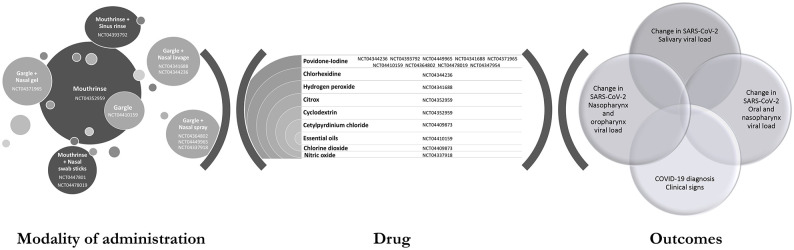
Current status of clinical trials on the use of mouth rinse for COVID-19 listed on the site ClinicalTrials.gov (Find Trials 2020).

Many articles have recommended the use of a mouth rinse to prevent the spread of SARS-CoV-2. However, to date (July 30), only 12 clinical protocols testing the effect of mouth rinse on SARS-CoV-2 are listed in ClinicalTrials.gov (Find Trials 2020; Fig. 1). One study used mouth rinse exclusively, 2 studies combined it with nasal swab sticks, and 1 study combined mouth rinse with a sinus rinse. Seven studies used mouth rinses as a gargle with or without nasal lavage, nasal spray, or nasal gel. Most of these trials evaluated PVP-I. The reduction of the salivary viral load, quantified using a polymerase chain reaction (PCR) technique, is a minority research objective. Indeed, only 3 studies had this objective. The focus of the other trials was mainly on the potential interaction of the viral load between the naso- and oropharynx with the combined use of gargle and nasal applications. Four trials are not yet recruiting participants, 6 are in the operational phase, and 2 have been completed but with only 18 and 20 participants included, respectively.

## Mouth Rinse Reagents with In Vitro or In Vivo Anti–SARS-CoV-2 Activity

### Mouth Rinses Containing PVP-I

PVP-I is composed of iodine and the water-soluble polymer polyvinylpyrrolidone. PVP-I has antimicrobial activity when it dissociates and releases iodine. Iodine penetrates the microbes, oxidizes nucleic acids, and disrupts proteins. Thus, PVP-I damages the virus via the perturbation of several metabolic pathways and disorganization of the cell membrane (Nagatake et al. 2002). PVP-I has been demonstrated to have greater antiviral activity against both enveloped and nonenveloped viruses (Table 2) as compared with other antiseptic agents, such as CHX (Kawana et al. 1997).

**Table 2.** Antiviral Activity of Reagents in Mouth Rinses.

	Antiviral Activity			Antiviral Activity against SARS-CoV-2		
Active Ingredient	In Silico	In Vitro	In Vivo	In Silico	In Vitro	In Vivo
Chlorhexidine	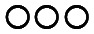	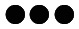	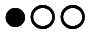	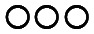	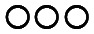	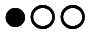
		CMV	HSV-1			
		FluV				
		HBV				
		HIV-1				
		HSV-1				
		Poliovirus				
		HCoV 229E				
Cetylpyridinium chloride	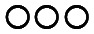	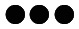	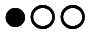	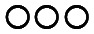	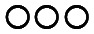	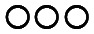
		HBV	Influenza A, B viruses			
		HSV-1				
		Influenza A, B viruses	Respiratory viruses			
Citrox	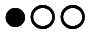	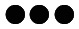	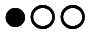	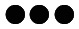	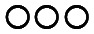	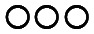
	HSV-1	Enterovirus A71	Influenza A virus			
		HBV				
		Influenza virus				
		RSV				
		Zika virus				
Cyclodextrin	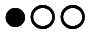	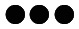	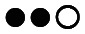	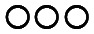	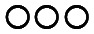	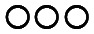
	Influenza A virus	Caprine	Influenza A virus			
		Parainfluenza virus				
		Type 3				
		EV-D68				
		HCV				
		Influenza A virus				
Essential oils	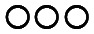	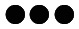	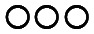	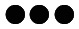	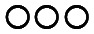	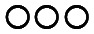
		Coxsackie virus				
		HAdV				
		HCMV				
		HIV				
		HSV-1-2				
		Influenza virus A (H1N1)				
		SARS-CoV				
		VSV				
		YF				
Hydrogen peroxide	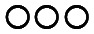	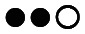	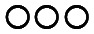	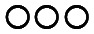	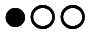	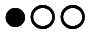
		Coronavirus				
		Influenza A, B viruses				
Povidone-iodine	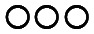	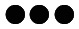	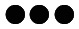	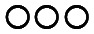	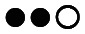	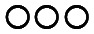
		Coxsackie virus, rhinovirus	Adenovirus			
		EBOV	Norovirus			
		HAdV				
		HIV				
		HPV				
		HRV				
		HSV-1				
		Influenza virus				
		Influenza virus A (H1N1)				
		Measles				
		MERS-CoV				
		Mumps				
		MVA				
		Poliovirus (1,3)				
		Polyomavirus				
		Rubella				
		SARS-CoV				

The electronic research was organized in the PubMed database. The following MeSH and non-MeSH search terms were used: (“Chlorhexidine” [MeSH terms] OR (“Cetylpyridinium chloride” [All fields] OR “essential oils” [All fields] OR “hydrogen peroxide” [All fields] OR “Povidone-iodine” [All fields] OR (“Citrox” [All fields] OR “Cyclodextrin” [All fields] AND (“Virucidal” [MeSH terms] OR (“Antiviral” [All fields]). The following codification was used: 5 articles or more listed in PubMed (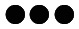), between 3 and 5 articles listed in PubMed (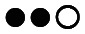), 1 or 2 articles listed in PubMed (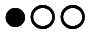), no articles listed in PubMed (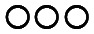). CMV, cytomegalovirus; EBOV, Ebola virus; EV-D68, enterovirus D68; H3N2 (FluV), human influenza virus A; HAdV, human adenovirus; HBV, hepatitis B; HCoV, human coronavirus; HCV, hepatitis C; HIV, human immunodeficiency virus type 1; HRV, human rotavirus; HSV-1, herpes simplex virus 1; MERS-CoV, middle east respiratory syndrome coronavirus; MVA, modified vaccinia virus Ankara; RSV, respiratory syncytium virus; SARS-CoV, severe acute respiratory syndrome coronavirus.

In vitro studies evaluating the 50% tissue culture infective dose (TCID50) method demonstrated that PVP-I has virucidal activity against SARS-CoV-2. Gargle and mouth rinse with solutions containing PVP-I at 1% achieved a virucidal activity higher than 99.99%, which corresponds to a reduction of virus load greater than 4 log_10_, after 30 s of contact (Anderson et al. 2020). These results are in accordance with those of Hassandarvish et al. (2020), who concluded that PVP-I 1% achieved a more than 5 log_10_ reduction in the virus titers after 15, 30, and 60 s of treatment. Applying 0.5% PVP-I for 15 s reduces the SARS-CoV-2 load by 4 log_10_, whereas application for 30 or 60 s reduces the load by more than 5 log_10_ (Hassandarvish et al. 2020). Bidra et al. (2020) observed inactivation of SARS-CoV-2 when applying PVP-I 0.5% for 15 s. The difference can be explained by the fact that the virus titers were calculated using a standard endpoint dilution of 50% cell culture infectious dose (CCID50).

In addition, previous studies have shown that the common use of PVP-I in mouth rinse has no deleterious health effects (Shiraishi and Nakagawa 2002). Type 1 allergy to PVP-I is considered to be rare (Lachapelle 2014). PVP-I can be safely used in the mouth at concentrations as high as 2.5% for up to 5 mo (Frank et al. 2020). Moreover, the topical application of PVP-I does not destroy the balance of the oral microbiota (Tsuda et al. 2020). However, the use of PVP-I is contraindicated in patients with an allergy to iodine, thyroid disease, pregnancy, or treatment with radioactive iodine (Gray et al. 2013).

In the interim guidelines for minimizing the risk of COVID-19 transmission, the American Dental Association recommends the use of a preoperative 0.2% PVP-I mouth rinse to decrease the risk of transmission of SARS-CoV-2 from the patient to the dentist (American Dental Association 2020). The use of PVP-I mouth rinse was also preconized by the Australian Dental Association and the Centers for Disease Control and Prevention (Australian Dental Association 2020; Centers for Disease Control and Prevention 2020). According to Challacombe et al. (2020), all patients requiring dental treatment should be administered a 0.5% PVP-I solution at a dose of 0.3 mL into each nostril, and 9 mL of the 0.5% solution should be used as a mouth rinse (30 s of distribution throughout the oral cavity and 30 s for gently gargling).

The action of mouth rinses containing PVP-I against SARS-CoV-2 will be due to the sensitivity of this virus to oxidation (Pattanshetty et al. 2020). In one recent communication that included 4 COVID-19 patients, the use of 15 mL of 1% PVP-I mouth rinse for 1 min significantly reduced the SARS-CoV-2 load, as evaluated by real-time reverse transcriptase PCR (rRT-PCR) in the saliva for 3 h (Martínez Lamas et al. 2020).

### Mouth Rinses Containing CHX

CHX is a cationic bisbiguanide used in general medical practice as a broad-spectrum antiseptic. CHX is known to have antiviral activity and is effective against lipid-enveloped viruses but not against nonenveloped viruses (Table 2; Bernstein et al. 1990). Thus, a recent review preconized its use in reducing the risk of spreading SARS-CoV-2 through aerosols, although its action against this virus remains controversial (Herrera et al. 2020). Conversely, the *Guidelines for the Diagnosis and Treatment of New Coronavirus Pneumonia* (5th edition) of the National Health Commission of the Republic of China explained that CHX as a mouth rinse may not be efficient at killing SARS-CoV-2 (Peng et al. 2020).

Although mouth rinses containing CHX are often used, only 1 study focused on the effect on SARS-CoV-2. Yoon et al. (2020) evaluated the viral load in the saliva of 2 COVID-19 patients from hospital day 1 to 9 by rRT-PCR. Moreover, on days 3 and 6, the patients used CHX mouth rinse (0.12%, 15 mL) for 30 s. The salivary load of SARS-CoV-2 was evaluated before gargling and after 1, 2, and 4 h. A transient decrease in the viral load was observed for 2 h postgargling, but it increased again after that. The main limitations of this study are the small number of subjects and the absence of controls (gargling with saline). Thus, if the results are confirmed by other clinical trials, CHX mouth rinses could help to prevent the spread of SARS-CoV-2.

### Mouth Rinses Containing H_2_O_2_

H_2_O_2_ is a chemical compound. It is a widely used antimicrobial, and its efficacy has been demonstrated on several human viruses, among which coronavirus and influenza viruses were found to be most sensitive (Table 2; Kumar et al. 2020). H_2_O_2_ targets the viral lipid envelope of these viruses and, more particularly, of SARS-CoV-2 (O’Donnell et al. 2020). It liberates oxygen-free radicals and disrupts the lipid membrane (Peng et al. 2020). H_2_O_2_ presents the advantage of being safe for mucous membranes whether used in mouth rinse or nasal spray, even when used at a concentration of 3% over 6 mo (Caruso et al. 2020).

A letter to the editor advised the off-label use of H_2_O_2_ at concentrations of 3% and 1.5% by oral and nasal washing, respectively (Caruso et al. 2020). The authors recommended rinsing the mouth 3 times per day and performing a nasal wash 2 times per day from the onset of the first symptoms and the presumptive diagnosis of COVID-19 and during the illness or by hospitalized patients not requiring intensive care. Even if the use of mouth rinses containing H_2_O_2_ before dental procedures is recommended by several associations, such as the American Dental Association (2020), only 1 in vitro study has been published, and no in vivo evidence exists to date (Ortega et al. 2020).

In their in vitro study, Brida et al. (2020) used the CCID50 method to evaluate the inactivation of SARS-CoV-2 with H_2_O_2_ mouth rinse and compared it with PVP-I mouth rinse (Bidra et al. 2020). To be in accordance with the clinically recommended concentrations, the concentrations tested were 0.5%, 1.25%, or 1.5% for PVP-I and 3% or 1.5% for H_2_O_2_. Although PVP-I completely inactivated SARS-CoV-2 after 15 and 30 s of contact, H_2_O_2_ showed minimal inactivation.

In a prospective, in vivo, clinical pilot study, Gottsauner et al. (2020) used RT-PCR to analyze the effect of gargling in the mouth and throat with 20 mL of 1% hydrogen peroxide for 30 s. No control group (e.g., a placebo mouth rinse without hydrogen peroxide) was included. Among the 10 COVID-19 patients included, there was no significant decrease in SARS-CoV-2 viral load.

## Mouth Rinse Reagents with In Silico Anti–SARS-CoV-2 Activity

### Mouth Rinses Containing Citrox

Citrox, which is derived from citrus fruits, is composed of soluble bioflavonoids and hydroxylated phenolic structures produced by plants. Bioflavonoids have demonstrated their capacity to act against bacteria, fungi, and viruses (Hooper et al. 2011; Lalani et al. 2020; Reis et al. 2020; Zou et al. 2020).

Although no in vitro or in vivo studies have been published on Citrox mouth rinse, in silico studies based on computer virtual screening predicted an antiviral action against SARS-CoV-2. Hu et al. (2020) targeted the SARS-CoV-2 main protease and host toll-like receptors (TLRs) to determine potential inhibitors. The citrus flavonoid rutin was the best candidate among the traditional antiviral medicinal plants. It can fit into the substrate-binding pocket of the SARS-CoV-2 main protease and interact with TLRs such as TLR2, TLR6, and TLR7, which affect the assembly and function of the viral protein and the host inflammatory response (Hu et al. 2020).

According to a docking analysis, hesperidin, a bioflavonoid contained in citrus peel, may bind to 3 protein receptors of SARS-CoV-2 responsible for cell infection and virus replication (SARS-CoV-2 protease domain, receptor-binding domain of the spike glycoprotein [RBD], and receptor-binding domain of the ACE2 at the protease domain; Meneguzzo et al. 2020). Based on these predictive results, it is likely that because of its binding affinity to these 3 main targets, hesperidin would fight the viral infection by inhibiting either virus binding to ACE2 or virus replication in cells. Wu et al. computed that hesperidin can interact with RBD, which disrupts the interaction of ACE2 with RBD and prevents SARS-CoV-2 from entering the cell (Wu et al. 2020). A further study screened the inhibitors of the 3-chymotrypsin–like protease of the SARS-CoV-2, a protein vital to virus replication. Hesperidin (an approved drug) and the flavonoid glycoside diosmin (a preapproved drug) inserted into and blocked the substrate binding site. Moreover, hesperidin had several modes of binding (Chen et al. 2020).

The modeling of other flavonoids, such as naringin, caflanone, equivir, hesperetin, myricetin, and Linebacker, forecasted high affinity to helicase, spike, and protease sites on the ACE2 receptor. This interaction would provoke a conformational change and the inhibition of the entry of SARS-CoV-2 (Meneguzzo et al. 2020; Ngwa et al. 2020). Moreover, nargintin and caflanone are also able to restrain the proinflammatory overreaction of the immune system (Meneguzzo et al. 2020; Ngwa et al. 2020).

### Mouth Rinses Containing EOs

EOs are volatile and odorous products extracted from the stems, leaves, flowers, bark, fruits, and roots of plants. Compounds of EOs are synthetized through the pathways of mevalonic acid, malonic acid, and methyl-d-erythritol-4-phosphate in the cytoplasm and plastids of plant cells. Even if EOs are mainly composed of 2 or 3 components that represent between 20% and 70% of their makeup, they are much more complex structures. The main compounds are terpenes, terpenoids, and phenylpropanoids, but other compounds, such as oxides, fatty acids, and sulphur derivatives, are present (Wińska et al. 2019). Several EOs have demonstrated antibacterial, antiviral (Table 2), antifungal, antioxidant, and anti-inflammatory properties (Wińska et al. 2019).

The EOs act before the addition or adsorption of the virus to cell monolayers (i.e., before the entry of virus into the cells). Indeed, EOs interfere with the phospholipid bilayer of coronaviruses, provoking the dislocation of the viral envelope (Wińska et al. 2019).

Several in silico studies predicted the antiviral effects of EOs against SARS-CoV-2. Seventeen compounds of garlic oil were predicted to interact with the viral main protease (Mpro/6LU7) of SARC-CoV-2 (Thuy et al. 2020). Another molecular docking analysis predicted that among the 171 screened EO compounds, (E,E)-α-farnesene, (E,E)-farnesol, and (E)-nerolidol may interact with SARS-CoV-2 Mpro, thereby inhibiting viral replication (Silva et al. 2020). Moreover, α-bulnesene, eremanthin, (E,E)-α-farnesene, (E)-β-farnesene, (E,E)-farnesol, (E)-nerolidol, β-sesquiphellandrene, and (Z)-spiroether may bind to human ACE2, and (E)-cinnamyl acetate, eremanthin, (E,E)-α-farnesene, (E)-β-farnesene, (E,E)-farnesol, and geranyl formate may interact with SARS-CoV-2 spike proteins (Silva et al. 2020). Based on 2 docking analyses (Elfiky and Ibrahim 2020; Kulkarni et al. 2020), Asif et al. proposed that cinnamaldehyde may block the attachment of SARS-CoV-2 (Asif et al. 2020). Moreover, docking scores revealed that eugenol, menthol, and carvacrol have binding affinity for SARS-CoV-2 spike protein, Mpro, RNA-dependent RNA polymerase, and human ACE2 proteins (Silva et al. 2020). Neither in vitro nor in vivo studies have been published concerning the antiviral effects of EOs against SARS-CoV-2.

## Mouth Rinse Reagents with Potential Anti–SARS-CoV-2 Activity

### Mouth Rinses Containing CPC

CPC or N-hexadecyl pyridinium chloride is a cationic quaternary ammonium compound that is soluble in water and in aqueous solutions, nonoxidant or corrosive, and highly cationic at neutral pH (Herrera et al. 2020). CPC has a broad antimicrobial spectrum with a rapid bactericidal effect on gram-positive pathogens and a fungicide effect on yeasts in particular. Moreover, CPC has revealed antiviral activity against several viruses, and particularly in the case of treatment against respiratory infections (Table 2), but its action against SARS-CoV-2 remains to be elucidated (Baker et al. 2020). Indeed, CPC inactivates the virus through its lysosomotropic action and by destroying the capsid (Baker et al. 2020).

Interestingly, CPC is considered to be “generally regarded as safe” by the Food Drug Administration. It is usually found in mouth rinses and is suggested to fight SARS-CoV-2 (Baker et al. 2020). Using a compound library, Shen et al. (2019) identified 56 compounds exhibiting antiviral activity against genetically engineered human CoV OC43 (HCoV-OC43). Of these, 36 were confirmed to be also effective against wild-type HCoV-OC43. Among these, CPC exhibited antiviral activity against severe CoV (MERS-CoV) and HCoV-NL63, with an EC_50_ value of <5 µM, which represents the value of a compound that was considered effective. CPC was rated as the ninth most relevant among the 36 compounds (Shen et al. 2019). However, its clinical efficacy remains to be explored.

### Mouth Rinses Containing CDs

CDs are cyclic molecules composed of α(1 to 4)–linked glucopyranoside units, which number 6 for α-CD, 7 for β-CD, and 8 for γ-CD (Braga 2019). Native CDs can be modified by adding functions on their scaffolds. However, only a few are approved for human use in the fields of pharmacy, including 2-hydroxypropyl-β-CD, 2-hydroxypropyl-γ-CD, the randomly methylated β-CD, or the sulfobutyl ether β-CD.

Based on computer virtual screening, CDs are known to be active against enveloped and nonenveloped viruses (Table 2; Braga 2019). CDs could act against SARS-CoV-2 by targeting its lipid bilayer or metabolism (Abu-Farha et al. 2020). Compared with the mechanism of action on other viruses, a positive virucidal action is expected. Effectively, as CDs are able to bind to and sequester cholesterol, and because SARS-CoV-2 has this biomolecule in the lipid rafts of its membrane, CDs could inhibit human SARS-CoV-2 entry into host cells, reducing the infectivity of viral particles.

CDs are generally considered safe for humans. No restrictions concerning the intake of α-CD and γ-CD have been observed. For the oral intake of β-CD, a maximum dose of 5 mg per kilogram of weight each day is recommended (Braga 2019).

### Ethanol Used as Excipient in Mouth Rinses

Ethanol is an excipient that may have a role to play in the fight against SARS-CoV-2 in addition to previous compounds. Although it is active at high concentration on the inactivation of enveloped viruses, it is available at the lowest concentrations in many mouthwashes with graduated formulations from 14% to 27% (w/v; O’Donnell et al. 2020). Ethanol acts on microorganisms by dissolving the lipid membrane and denaturing proteins (Jing et al. 2020). In their in vitro study, Bidra et al. demonstrated, using the CCID50 method, that 70% ethanol was able to inactivate SARS-CoV-2 at 30 s of contact but was unable to completely inactivate the virus after 15 s of contact (Bidra et al. 2020). Thus, the impact of less toxic concentrations of ethanol is not scientifically established. This should be evaluated in vitro and in vivo as part of the potential role of mouthwashes against SARS-CoV-2.

## Conclusion

Until now, there has been no scientific evidence to recommend mouth rinses with anti–SARS-CoV-2 effect to control the viral load in the oral cavity. Some ingredients in antiseptic mouth rinses have antiviral properties, which could decrease the SARS-CoV-2 viral load of droplets emitted by COVID-19 patients. Because only a few in vivo, in vitro, and in silico studies has been conducted as of September 2020, there is also not sufficient scientific evidence to support the recommendation to manage the risk of infection in the dental office and in the community. In the meantime, the WHO’s preventive measures remain strongly recommended: wearing masks, washing hands, ventilating the premises, and maintaining social distance. A clinical trial of the potential applications of existing mouth rinses is therefore essential. In addition, in the near future, the results of clinical studies already planned should contribute to a better understanding of the anti–SARS-CoV-2 activities of the active ingredients.

## Author Contributions

F. Carrouel, D. Bourgeois, contributed to the conception, design, data acquisition, and interpretation, drafted the manuscript. L.S. Gonçalves, M.P. Conte, G. Campus, J. Fisher, L. Fraticelli, E. Gadea-Deschamps, L. Ottolenghi, contributed to data acquisition and interpretation, critically revised the manuscript.
